# The Comparison of Sun Protection Factor 30 Persistence Between Inorganic and Organic Sunscreen in Swimmers: Double-blind Randomized Clinical Trial

**DOI:** 10.2196/41633

**Published:** 2023-01-04

**Authors:** Karin Rachmani, Shannaz Nadia Yusharyahya, Adhimukti Sampurna, Respati W Ranakusuma, Sandra Widaty

**Affiliations:** 1 Department of Dermatology and Venereology, Faculty of Medicine Universitas Indonesia Cipto Mangunkusumo General Hospital Jakarta Indonesia; 2 Clinical Epidemiology and Evidence-Based Medicine Unit, Faculty of Medicine Universitas Indonesia Cipto Mangunkusumo General Hospital Jakarta Indonesia

**Keywords:** inorganic sunscreen, organic sunscreen, outdoor pool, sun protection factor, swimmer, sun protection, sunscreen, sun exposure, sunburn, prevention, efficacy, skin care, dermatology

## Abstract

**Background:**

Long-term sun exposure is one of the risks faced by outdoor swimmers and can cause sunburn. Using sunscreen is one way to prevent sunburn; however, physical activity can trigger sweat, friction, and water washing that can interfere with sunscreen efficacy and decrease its sun protection factor (SPF). Sunscreens are classified into inorganic and organic based on their filter. Organic sunscreen has a better bond to the skin than inorganic sunscreen, which forms a barrier above the skin layer that makes removing it easier. Organic sunscreen lasts longer than inorganic sunscreen when used in physical activities, but it has a limited spectrum, is more photolabile, and is more allergenic.

**Objective:**

This study aims to evaluate the persistency of SPF 30 between inorganic and organic sunscreens on the back area after 1.5 hours of swimming.

**Methods:**

This study is a randomized, split-body, double-blind clinical trial to evaluate the persistency of SPF 30 of the inorganic versus organic sunscreens in swimmers. Randomization was done to allocate the participants into treatment groups. Each participant received inorganic and organic sunscreen treatments applied to the back area. The research participants were swimmers from the Cikini swimming pool and Bina Taruna swimming pool, both in Jakarta, Indonesia.

**Results:**

A total of 22 swimmers were enrolled in this study. The analysis showed no significant difference between the SPF of the two sunscreens before swimming (*P*=.22). After swimming, the SPF levels of both sunscreens decreased: the inorganic sunscreen decreased from a median of 27 (range 23-47) to 12.3 (range 8-19), and the organic sunscreen decreased from a median of 30 (range 24-47) to 9.9 (range 6-19), which was statistically significant (*P*<.001). When comparing the SPF of inorganic and organic sunscreens after swimming, there was a statistically significant difference in the decrease in SPF levels between the two groups (*P*=.02), which indicated a better SPF persistence for inorganic sunscreens when compared to organic sunscreens.

**Conclusions:**

There was a decrease in the SPF levels of inorganic and organic sunscreens after 1.5 hours of swimming, with better persistence in inorganic sunscreens compared to organic sunscreens.

**Trial Registration:**

ClinicalTrials.gov NCT04618536; https://clinicaltrials.gov/ct2/show/NCT04618536

**International Registered Report Identifier (IRRID):**

RR2-10.2196/42504

## Introduction

In Indonesia, swimmers commonly train about five times a week for 1.5 hours per day at outdoor or indoor pools. Training occurs in the morning or evening, where the UV index usually ranges from 1 to 4 [1]. Swimmers who train in outdoor pools are exposed to humidity, hot and cold weather, windy conditions, and long-term sun exposure or UV radiation [2]. These exposures can cause various skin disorders such as sunburn [3,4].

For sunburn prevention, protection from the sun is needed and can be achieved in several ways, one of which is by using sunscreen [5,6]. There are two types of sunscreens based on their filters, specifically, organic and inorganic. Organic sunscreen absorbs and prevents UV light from entering the epidermis. Meanwhile, inorganic sunscreen works by reflecting and scattering radiation [5,7,8]. Previous trials have shown that organic sunscreens had better bonding properties to the skin layer than inorganic sunscreens. Meanwhile, inorganic sunscreens tend to form layers on the skin’s surface so they can be removed easily [7,8]. This study aimed to evaluate the persistence of sunscreen with sun protection factor (SPF) 30 used by swimmers after 1.5 hours of training.

## Methods

### Ethics Approval

This clinical trial has received ethics approval from the Ethical Committee Faculty of Medicine Universitas Indonesia (ID 20-09-1037).

### Study Design

This randomized, split-body, double-blind, noninferiority, and multicenter clinical trial was done from August to December 2020. This clinical trial has been registered into ClinicalTrials.gov with the identifier NCT04618536. The primary objective of this clinical study is to compare the persistency of the SPF between inorganic and organic sunscreens after 1.5 hours of swimming in the athlete population. The difference in the SPF before and after swimming was measured and compared. The SPF was quantified using a minimal erythema dose (MED) test conducted over 2 days. Irradiation was conducted on the first day, and 24 hours after irradiation, the result was collected. Furthermore, we assessed the SPF value based on the in vivo method conducted before swimming using an MED as the primary objective. This trial also aims to know the decreased level of SPF after swimming for 1.5 hours and which type of sunscreen provides higher persistence.

### Recruitment

Swimmers were recruited from Millenium Aquatic swimming club, which practice at the Cikini swimming pool, and Bina Taruna swimming club, which practice at the Bojana Tirta swimming pool, both in Jakarta, Indonesia.

### Participants

The inclusion criteria of this study were as follows:

- Female or male swimmers aged 18-40 years who practice swimming at least three times a week for 1.5 hours in the morning or evening
- Willing to consent to being a research participant
- Does not have skin diseases
- Does not have a history of allergies to sunscreens

Conversely, the exclusion criteria applied for this study were as follows:

- The existence of skin lesions in the test area
- Undergoing phototherapy treatment
- Using drugs with photosensitivity as a side effect
- Having a history of malignancy
- Showing photosensitivity reactions or disease affected by UV rays, direct sunlight exposure to the test area within 24 hours before the study, or during the study period
- Absence of erythema response 24 hours after the radiation test
- Erythema that occurs in the entire test area box within 24 hours after the radiation test

### Randomization and Masking

Computer-based randomization was done to allocate the participants into treatment groups. The split-body method was conducted on the same person to collect the data. Each participant received both inorganic and organic sunscreens simultaneously on the right or left (by random) of the back area. Treatment was allocated by numbering the research participants (1 being the right back area and 2 being the left back area). We used a computer-based randomization method to determine which side of the back area and type of sunscreen should be given [9]. The allocation data for each participant was placed in a sealed opaque envelope. Both the research participants and the researchers, but not the statistician, were unaware of the type of sunscreen that was be applied. The research assistant applied the sunscreen. Researchers were responsible for carrying out the irradiation test and assessment. At the end of data collection, a randomization code was revealed by a statistician who was not involved in the sampling process.

### Sunscreen

This study used inorganic and organic sunscreens made by PT Paragon Technology and Innovation with the formulation adjusted according to the research needs. Both sunscreens are made in the form of oil in water emulsion with the addition of a film-forming layer. The inorganic and organic sunscreens have the same base ingredients but different filters: titanium dioxide and zinc oxide for inorganic sunscreen, and diethylamino hydroxybenzoyl hexyl benzoate, tris-biphenyl triazine, ethylhexyl triazone, ethylhexyl salicylate, and methylene bis-benzotriazolyl tetramethylbutylphenol for the organic sunscreen.

### Procedure

**Figure 1:**
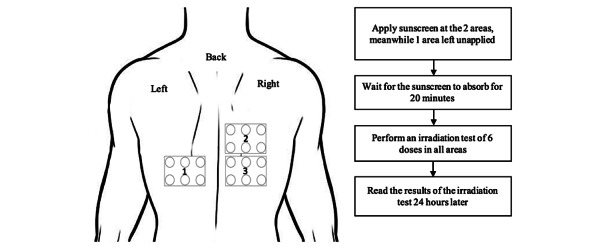
Procedure of research at the first session.

**Figure 2:**
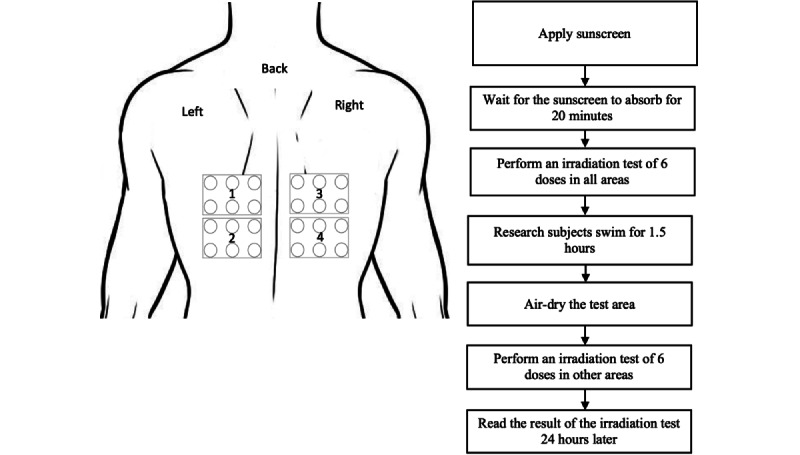
Procedure of research at the second session.

A COVID-19 prevention protocol was carried out to prevent the spread of the pandemic by using masks, face shields, handwashing, general physical examination, and social distancing of at least 1 meter during data collection. The general cleaning of tools and tool calibration were done for each research participant. Before data collection began, we conducted a preliminary study to identify the value of broadband UV-B (BB-UVB) MED on various skin types and interreviewer reliability tests to ensure the production of high-quality data during research. After the consent was obtained from the research participants, we performed history taking, physical examination, and documentation. Two sessions were held 1 week apart: the first session for primary data collection (skin type and identification of any skin lesion) and the second session for randomizing the participant and providing the treatment as stated in Figures 1 and 2.

At the first meeting, we marked the back of the swimmers with three areas. At the second meeting there were four areas marked. Each area is 40 cm^2^ and marked with a perforated sticker. The sunscreen was applied at a dose of 2 mg/cm^2^ using a 1 cc syringe for each area. Afterward, the sunscreen was spread using gloves, starting with circular and then followed by horizontal and vertical movements with light pressure for 35 seconds. The irradiation test was done 20 minutes after sunscreen application, using a metal halide UV-enhanced lamp BB-UVB in the active spectrum of 290-320 nm (The Daavlin Lumera). The MED values were calculated 24 hours after the test. The SPF of each sunscreen was compared before and after the swimming period. Swimming activities occurred in the morning or evening when the UV index was in the 0-2 range. Sunscreens used in this study were creams (oil in water emulsion) with a film-forming layer. Sunscreens were made by PT Paragon Technology and Innovation. If there were any severe side effects, the study would have been discontinued, such as anaphylactic reactions. Research participants who experienced any side effects were excluded from the study, but their development will be followed until they recover.

### Statistical Analysis

The minimum estimated sample size was calculated using the difference in the average decrease in SPF levels that is considered significant based on the clinical judgment set by the researchers as 5. A total of 22 participants in the experimental and control groups were estimated to be needed to reject the null hypothesis that the population means of the experimental and control groups are equal, with a probability (power) of 0.9. The type I error probability associated with this null hypothesis test is 0.05. The collected data were analyzed using SPSS version 20.0 (IBM Corp) software in descriptive and inferential analysis. The persistency of the organic and inorganic sunscreens’ SPF was assessed using the MED, stated in mJ/cm^2^. The SPF was calculated from the ratio between MED in protected and unprotected skin areas and persistence of SPF, stated in index units. The persistency of SPF was defined as the lowest differences of SPF before and after 1.5 hours of swimming. We used a 1-sided CI approach in the statistical methods. The mean difference in SPF will be no different if the *P* value for the paired *t* test or Wilcoxon test is >.05 and the upper limit of the CI does not exceed 4 SPF.

## Results

**Figure 3:**
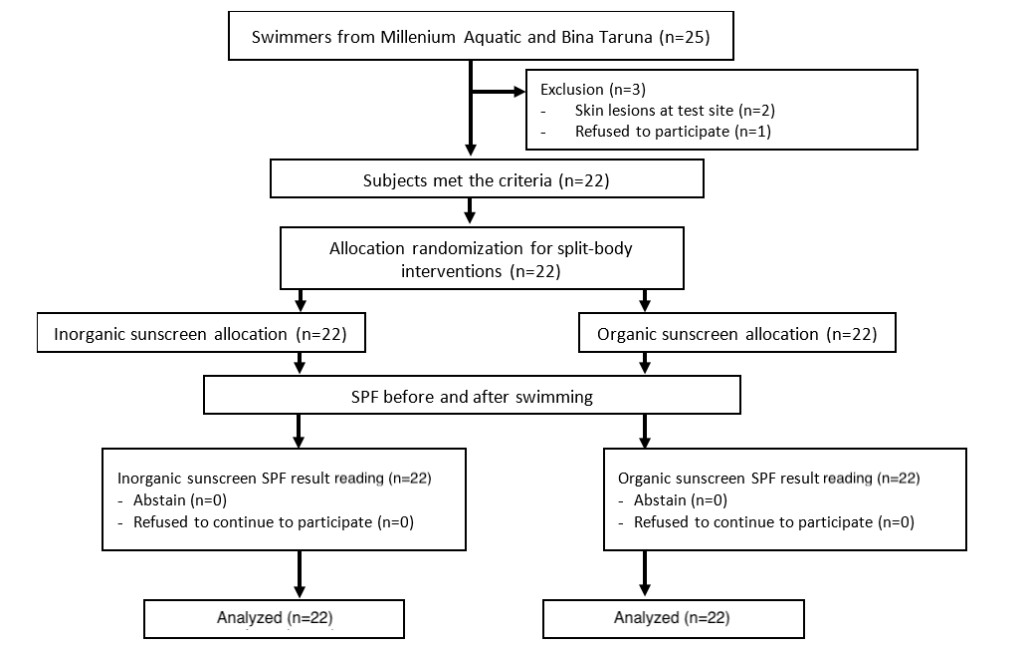
The flowchart for participant enrollment, assignment, allocation, follow-up, and analysis for the split-body interventions. SPF: sun protection factor.

Initially, the research was planned to include only Millenium Aquatic swimmers from one swimming pool, namely, the Cikini swimming pool. However, some research participants could not attend the sampling session due to the pandemic conditions, so we added swimmers from the Bina Taruna swimming club who practice at another location, namely, the Bojana Tirta swimming pool. This research took place from August to December 2020, and the enrollment of the participants ended when the minimum sample size was achieved. Of the 25 swimmers from both swimming clubs, 22 were included in the study based on the inclusion and exclusion criteria. A total of 14 swimmers came from the Millenium Aquatic swimming club and 8 from the Bina Taruna swimming club (Figure 3).

This study’s demographic distribution showed that participants were mostly male, had Fitzpatrick skin types III and IV, were unmarried, and had an undergraduate education. The median age of participants was 22 (range 19-28) years, and 64% (n=14) of the participants were Millenium Aquatic members (Table 1). Swimmers had an average training session six times per week starting at 6 AM (based on Jakarta, Indonesia time). As many as 27% (n=6) of the participants had a history of sunburn when exposed to UV radiation. We also analyzed the temperature, pH, osmolarity, and conductivity differences between the two swimming pools (Table 2).

**Table 1:** Clinical characteristics of research participants (N=22).

Characteristics		Values		
Age (years), median (range)		22 (19-28)		
**Gender, n (%)**				
	Male	12 (54)		
	Female	10 (46)		
**Swimming club, n (%)**				
	Millenium Aquatic	14 (64)		
	Bina Taruna	8 (36)		
**Marital status, n (%)**				
	Yes	1 (5)		
	No	21 (95)		
**Education, n (%)**				
	High school	10 (46)		
	Undergraduate	12 (54)		
**Skin type, n (%)**				
	Type III	13 (59)		
	Type IV	9 (41)		
**MED^a^ based on skin type (mJ/cm^2^), mean (SD)**				
	Type III	107.23 (20.51)		
	Type IV	131.33 (29.38)		
**MED based on swimming club (mJ/cm^2^), mean (SD)**				
	Millenium Aquatic athletes	106.86 (19.28)		
	Bina Taruna athletes	135 (29.70)		
**Frequency of swimming practice per week, n (%)**				
	Less than 6 times	10 (46)		
	More than 6 times	12 (54)		
**History of skin disease, n (%)**				
	Yes	4 (18)		
	No	18 (82)		
**History of skin cancer, n (%)**				
	Yes	0 (0)		
	No	22 (100)		
**History of allergies, n (%)**				
	Yes	4 (9)		
	No	18 (91)		
**History of red skin after sun exposure, n (%)**				
	Yes	6 (27)		
	No	16 (73)		
**History of skin complaints after sun exposure, n (%)**				
	Yes	5 (23)		
	No	17 (77)		
**Current skin disease, n (%)**				
	Yes	3 (14)		
	No	19 (86)		
**History of drug consumption, n (%)**				
	Yes	0 (0)		
	No	22 (100)		
**Sport beside swimming, n (%)**				
	Yes	9 (41)		
	No	13 (59)		

^a^MED: minimal erythemal dose.

**Table 2:** Swimming pool profiles.

	Cikini swimming pool	Bojana Tirta swimming pool
Temperature (°C), mean (SD)	28.5 (0.6)	29.4 (0.9)
pH, mean (SD)	8.4 (0.3)	7.6 (0.2)
Osmolarity (ppm), median (range)	469 (105-506)	187.5 (180-195)
Conductivity (μS/cm), median (range)	952.5 (404-1013)	432 (390-455)

After swimming, the SPF levels decreased in both sunscreens, namely, the SPF of the inorganic sunscreens decreased from a median of 27 (range 23-47) to 12.3 (range 8-19), and the SPF of the organic sunscreens decreased from a median of 30 (range 24-47) to 9.9 (range 6-19; Table 3). The decrease in the SPF levels in each group of sunscreens was statistically significant (*P*<.001). Based on the analysis results, there was no significant difference in the SPF of the two sunscreens on the research participants before swimming. When we compared the SPF of the inorganic and organic sunscreens after swimming, there was a difference in the decrease in SPF levels between the two groups. This difference was statistically significant (*P*=.02) and indicated that the inorganic sunscreen had a better SPF persistence than the organic sunscreens (Table 4). No serious adverse events or other harms were encountered during this study.

**Table 3:** Comparison of sun protection factor (SPF) in each sunscreen group after swimming.

	Before swimming, median (range)	After swimming, median (range)	*P* value
SPF of inorganic sunscreen^a^	27 (23-47)	12.3 (8-19)	<.001
SPF of organic sunscreen^a^	30 (24-47)	9.9 (6-19)	<.001

^a^Data analyzed using Wilcoxon test.

**Table 4:** Comparison of sun protection factor (SPF) between inorganic and organic sunscreen before and after swimming.

	Type of sunscreen (SPF), median (range)		*P* value
	Inorganic	Organic	
Before swimming^a^	27 (23-47)	30 (24-47)	.22
After swimming^a^	12.3 (8-19)	9.9 (6-19)	.02

^a^Data were analyzed using Wilcoxon test.

## Discussion

### Principal Results

The participants of this study had a median age of 22 years and consisted of more male swimmers (n=12, 54%) than female swimmers (n=10, 46%). Differences in age, height, and weight determined the variability of distance, style, and time of the swimmers. The participants in this study had similar ages to other studies, namely, the average age of female swimmers compared to male swimmers was 22.7 and 23.2 years, respectively [10]. A cross-sectional study in the United States in 2016 and 2017 showed that sunburn was significantly higher in young adults (aged 18-29 years; *P*<.001), especially for those with a previous history of sensitive skin [11]. A study conducted on 246 participants in Spain, involving various athletes from water sports aged 16-30 years showed no significant difference between the sexes regarding habits for sun protection [12]. Qualitative research has stated that women use sunscreen more often and protect themselves from the sun more than men because they have a higher knowledge about skin cancer and feel more at risk of developing cancer. Men tend to think of using sunscreen only when they are at the beach, while women think sunscreen is a daily necessity and must be applied before leaving the house [13].

The number of participants who practiced in the Cikini swimming pool (n=14, 64%) was more than in the Bojana Tirta swimming pool (n=8, 36%). The frequency of exercise was generally more than six times per week for 54% (n=12) of the participants. Almost all research participants started swimming exercises before 8 AM (Jakarta, Indonesia time). A total of 54% (n=12) of the participants had a bachelor’s degree. The Fitzpatrick skin type of the research participants was mostly type III and type IV. A history of skin diseases and allergies was rarely found in the study participants. None of the study participants had a history of skin cancer or routine drug use. More than 70% of the research participants had no complaints after sun exposure. More than 50% of the research participants did sports other than swimming, such as running, cycling, futsal, and soccer. These findings suggest an increased risk of greater exposure to UV radiation for swimmers.

The characteristics of the water in swimming pools, such as pH, temperature, conductivity, and osmolarity, play a role in influencing water quality. Based on the World Health Organization recommendations, swimming pools must have specific physicochemical parameters to ensure that the water does no harm. Our findings show that both swimming pools were in accordance with the guideline [14,15].

Fitzpatrick divides skin type based on the response to sun exposure, namely, burning and tanning. Influencing factors of this skin type include genetic predisposition and habits that increase UV radiation exposure, sunbathing activities, and the use of sunscreen [16]. This study showed that MED was greater in skin type IV after 24 hours of exposure to unprotected skin than type III. The MED values were higher in skin types IV-VI because of the higher levels of eumelanin, which makes it appear darker. Low MED values in skin types I-III cause the skin to be prone to inflammation, be more sensitive, burn easily, and increase the risk of skin cancer [17]. Another study showed a strong correlation between the MED and Fitzpatrick skin type (correlation coefficient 0.5-0.69). However, the discrimination value of Fitzpatrick’s skin type is poor, which can lead to different skin types having the same MED value [18].

Sunscreens are classified according to their filter, namely, organic and inorganic. Organic sunscreens change the conformation of molecules to prevent UV radiation from reaching the skin, while inorganic sunscreens reflect and scatter light [19]. This study used diethylamino hydroxybenzoyl hexyl benzoate, tris-biphenyl triazine, ethylhexyl triazone, ethylhexyl salicylate, methylene bis-benzotriazolyl tetramethylbutylphenol, and bis-ethylhexyloxyphenol methoxyphenyl triazine as organic filters. These components are photostable and well-dispersed oil-soluble filters in aqueous phase emulsions, producing higher SPF values. This can be seen from the organic SPF value of the sunscreen when it has not been used for swimming [5].

Inorganic filters used in this study were titanium dioxide and zinc oxide. Both are metal oxide particles that have long been used as filters in sunscreens and are efficient and photostable. Because both are metal oxide particles, these filters must be coated with an inert substance. Silicon dioxide, dimethicone, and triethoxycaprylysilane were used as an inert substance to coat the inorganic filter. This coating aims to stabilize the titanium dioxide and zinc oxide so that they do not react when exposed to UV [5,20]. The use of silicone and its derivatives in this sunscreen can increase the resistance of emulsion preparations due to their hydrophobic nature [21,22]. This study used sunscreen in the form of cream (oil in water emulsion) with the addition of a film-forming agent. Isododecane and trimethylsiloxysilylcarbomoyl pullulan are film-forming materials based on silicone polymers. Polymers provide a water-resistant effect, contributing to an increase in SPF levels, and affect the sensory effects of sunscreen formulations. The purpose of adding polymers is to increase water resistance and increase SPF levels. The addition of polymers will add a layer on top of the sunscreen in the form of a hydrophobic barrier so that the sunscreen layer cannot bind to water and becomes more challenging to wash off. The hydrophobic and water-resistant sunscreen layers that adhere to the skin’s surface have the potential to withstand constant hygroscopic pressure and help maintain the integrity of the skin barrier. This underlies the resistance of both sunscreens in the study after swimming [23-25].

### Limitations

The data collection was initially planned in one swimming pool. However, due to the COVID-19 pandemic, a limited number of swimmers were available, which resulted in an insufficient sample size. We had to add more swimmers who practiced at another swimming pool. It is assumed that the effectiveness of the sunscreens has been affected due to the differences in the characteristics of the two swimming pools, namely, the temperature, pH, osmolarity, and conductivity.

### Comparison With Prior Work

This research is the first double-blind randomized clinical trial to compare sunscreen filters under actual conditions in skin types III and IV. Based on our literature review, there has been no study like this before. Hopefully the research results can be used to develop further inorganic sunscreens for swimming or other sports.

### Conclusions

In conclusion, there was a decrease in the SPF levels of inorganic and organic sunscreen after swimming for 1.5 hours, whereas the SPF persistence of the inorganic sunscreen was better than the organic sunscreen.

## Appendix

Multimedia Appendix 1 CONSORT checklist.

## Abbreviations

BB-UVB: broadband UV-B
MED: minimal erythema dose
SPF: sun protection factor
